# A call to action; an open letter to WHO from the international interventional radiology community

**DOI:** 10.1186/s42155-020-00195-2

**Published:** 2021-01-04

**Authors:** Yi Yang, Andrew Moore, Fabian Laage Gaupp, Rakesh Ahuja, Charles Sanyika, Gregory C Makris

**Affiliations:** 1 Department of Radiology, Aventura Hospital and Medical Center, Aventura, Florida USA; 2 Department of Radiology, Integris Baptist Medical Center, Oklahoma City, USA; 3 Vascular and Interventional Radiology Department, Yale New Heaven Medical Center, Connecticut New Haven, USA; 4 Department of Radiology, Einstein Medical Center, Philadelphia, USA; 5 Department of Radiology, Donald Gordon Medical centre, Johannesburg, South Africa; 6 Vascular and Interventional Radiology Department, Guy’s and St Thomas’ Hospital, NHS Foundation Trust, London, UK; 7 Alfa Institute of Biomedical Sciences, Neapoleos 9, Marousi, Athens, Greece

**Keywords:** Interventional radiology, Global health

Dear Editor,

We appreciate the opportunity to share below our open letter to the World Health Organization (WHO). The intent of the letter is to initiate a discussion on and collaboration in addressing the dramatic lack of interventional radiology services in low income countries and the lack of associated data. This was a joined effort from the residents and fellows societies of the Society of Interventional Radiology, (SIR) USA, the European Trainee Forum of the Cardiovascular and Interventional Radiology Society of Europe (CIRSE) and the Society of African Interventional Radiology & Endovascular Therapy (SAFIRE).

Dear WHO,

Compared to the 1918 influenza and 2003 SARS-CoV epidemics we have progressed substantially as a responsive and connected global community. Prompt publication of countless dedicated research articles, public education through news outlets and social media on preventive measures, and initiation of vaccine development within weeks of the COVID-19 outbreak highlight the many benefits of global connectivity and collaboration. Yet, these times of fear and stress reveal the persistent chasm of racial inequality, healthcare inequity, and social injustice that have plagued humanity for centuries. These inequalities extend into almost every facet of medicine, but nowhere is the disparity more extreme than in minimally invasive treatment options available to patients in high-income countries and the near-complete lack thereof in most low- and middle-income countries (LMICs). Minimally invasive techniques used in interventional radiology (IR) can take a prominent role in achieving the Triple Billion Targets and Sustainable Development Goals (SDGs) of decreasing maternal mortality, road traffic mortality, non-communicable disease mortality, and healthcare expenditure. There are several hurdles to the implementation of IR in LMICs, including the sparsity of baseline data, limited access to equipment, and lack of training. In order to facilitate effective IR training and adequate supply of IR equipment, there is a critical need for improving and updating the “WHO Global Atlas of Medical Devices”.

The overall risk of perioperative mortality in Africa remains three times higher than in high-income countries and the risk of maternal death after cesarean section may be up to 100 times higher (Biccard et al. 2020; Sobhy et al. 2019; Makris and Byrne 2019). IR provides image-guided, minimally invasive procedures that have revolutionized modern medicine over the past half century (Baum and Baum 2014). These procedures decrease individual and societal costs by lowering complication rates, shortening hospital stay, and in some cases by opening up completely new avenues of treatment for cancer, infection, vascular disease, trauma, and peripartum complications. An additional advantage of these procedures is the decreased need for general anesthesia, as most image-guided procedures can be performed under sedation. Given the high surgical and anesthesia-related complication and mortality rates in LMICs we expect the many proven advantages of IR to be even more pronounced and provide even greater relative improvement of outcomes than in high-income countries. While early efforts to implement IR in Kenya, Nigeria, and Tanzania are promising, there are several roadblocks hindering more rapid expansion and implementation in other African nations, the most important of which is the lack of baseline information regarding the status of imaging equipment and academic training programs (Kline et al. 2017; Laage Gaupp et al. 2019).

The 2017 “WHO Global Atlas of Medical Devices” does not assess several medical device and equipment categories that are integral for diagnosis, treatment, and follow-up of patients by minimally invasive means. Here, we present what we believe should be included in future iterations in order to lay the ground for further expansion and adaptation of IR, especially in LMICs:

1. Ultrasound: As a non-ionizing imaging modality, ultrasound is the ideal first-line imaging tool in obstetrics, pediatrics, trauma triage, and is essential for breast cancer diagnosis, biopsy, and follow-up. Ultrasound is used in over 90% of IR procedures to guide percutaneous biopsies, vascular access, and drainage procedures.
2. Fluoroscopy: As a dynamic, X-ray based, real-time imaging modality, fluoroscopy is the other most essential imaging tool used in IR. With fluoroscopy, interventional radiologists can navigate wires and catheters to almost any place in the body in order to perform embolizations in the setting of hemorrhage, limb salvage from peripheral artery disease, extract clot in the setting of stroke or pulmonary emboli, and administer radio- and chemotherapy locally. These procedures are often life-saving and significantly less invasive than their surgical alternatives.
3. Systems for Reviewing Imaging: Determining the availability of PACS (Picture Archiving and Communication System), EMR (Electronic Medical Record), and/or access to a viewing box or written medical records which are paramount for initial evaluation and follow-up of the patient’s medical history, management planning, and disease status over time.

The availability of these data measures will clarify the global distribution of diagnostic and therapeutic imaging and procedural devices. This will allow IR societies, academic institutions, researchers, educators, and NGOs to strategize and prioritize where and how to best extend and implement minimally-invasive life-saving procedures in LMICs. Additionally, this data will facilitate industry growth and partnerships within the unique context of the LMIC setting.

We believe that increasing the robustness of the “WHO Global Atlas of Medical Devices” will contribute to increasing access to minimally-invasive procedures in LMICs. We want to be actively involved in this process to support the efforts of the WHO in this matter. For example, as trainees, we can assist with facilitating coordination with consultants and suppliers and lead research efforts in evaluating and guiding implementation. We appreciate your efforts in leading equity in global health and look forward to moving forward in our collective commitment to providing all patients with the care that they need and deserve.

## Data Availability

Data sharing not applicable to this article as no datasets were generated or analysed during the current study.
